# A Single Dose of an MVA Vaccine Expressing a Prefusion-Stabilized SARS-CoV-2 Spike Protein Neutralizes Variants of Concern and Protects Mice From a Lethal SARS-CoV-2 Infection

**DOI:** 10.3389/fimmu.2021.824728

**Published:** 2022-01-27

**Authors:** Patricia Pérez, Adrián Lázaro-Frías, Carmen Zamora, Pedro J. Sánchez-Cordón, David Astorgano, Joanna Luczkowiak, Rafael Delgado, José M. Casasnovas, Mariano Esteban, Juan García-Arriaza

**Affiliations:** ^1^ Department of Molecular and Cellular Biology, Centro Nacional de Biotecnología (CNB), Consejo Superior de Investigaciones Científicas (CSIC), Madrid, Spain; ^2^ Centro de Investigación Biomédica en Red de Enfermedades Infecciosas (CIBERINFEC), Madrid, Spain; ^3^ Pathology Department, Centro de Investigación en Sanidad Animal (CISA), Instituto Nacional de Investigación y Tecnología Agraria y Alimentaria (INIA), Consejo Superior de Investigaciones Científicas (CSIC), Madrid, Spain; ^4^ Instituto de Investigación Hospital Universitario 12 de Octubre (imas12), Madrid, Spain; ^5^ Department of Medicine, School of Medicine, Universidad Complutense de Madrid, Madrid, Spain; ^6^ Department of Macromolecular Structures, Centro Nacional de Biotecnología (CNB), Consejo Superior de Investigaciones Científicas (CSIC), Madrid, Spain

**Keywords:** SARS-CoV-2 vaccine, MVA vector, prefusion-stabilized S protein, protective efficacy, mouse model

## Abstract

We generated an optimized COVID-19 vaccine candidate based on the modified vaccinia virus Ankara (MVA) vector expressing a full-length prefusion-stabilized SARS-CoV-2 spike (S) protein, termed MVA-CoV2-S(3P). The S(3P) protein was expressed at higher levels (2-fold) than the non-stabilized S in cells infected with the corresponding recombinant MVA viruses. One single dose of MVA-CoV2-S(3P) induced higher IgG and neutralizing antibody titers against parental SARS-CoV-2 and variants of concern than MVA-CoV2-S in wild-type C57BL/6 and in transgenic K18-hACE2 mice. In immunized C57BL/6 mice, two doses of MVA-CoV2-S or MVA-CoV2-S(3P) induced similar levels of SARS-CoV-2-specific B- and T-cell immune responses. Remarkably, a single administration of MVA-CoV2-S(3P) protected all K18-hACE2 mice from morbidity and mortality caused by SARS-CoV-2 infection, reducing SARS-CoV-2 viral loads, histopathological lesions, and levels of pro-inflammatory cytokines in the lungs. These results demonstrated that expression of a novel full-length prefusion-stabilized SARS-CoV-2 S protein by the MVA poxvirus vector enhanced immunogenicity and efficacy against SARS-CoV-2 in animal models, further supporting MVA-CoV2-S(3P) as an optimized vaccine candidate for clinical trials.

## Introduction

Severe acute respiratory syndrome coronavirus 2 (SARS-CoV-2) emerged in December 2019 as the causative agent of coronavirus disease 2019 (COVID-19) (1, 2). The virus is highly transmissible among humans and it has spread rapidly. The unprecedented scale and severity of the COVID-19 pandemic prompted the rapid development of diagnostic tests, treatments, and, specially, vaccines that are contributing to reduce the incidence of the virus and its associated mortality. However, the number of human infections continues to be unabated due to the fact that, along the emergence of new variants with enhanced transmissibility, the vaccines have not reached most of the population (3). In addition, vaccine-elicited immune responses are not durable; hence, revaccination programs are being considered to keep and extend the longevity of immune responses. From the time when the pandemic was declared up till now, vaccine candidates based on different platforms (nucleic acids, proteins, non-replicating live virus vectors, and inactivated SARS-CoV-2 virus, among others) have been developed. Some of them are being used in immunization programs while others are at different stages of ongoing preclinical and clinical trials (https://covid19.trackvaccines.org/vaccines/). In Europe, the only four approved vaccines are based on mRNA (Moderna and Pfizer/BioNTech) or adenovirus vectors (AstraZeneca and Janssen), with high efficiency to control virus infection and COVID-19 disease (4–6). Whereas these vaccines represent a major advance, none of them have triggered sterile immunity, and hence, vaccinated individuals, when infected, can transmit the virus as recent studies have demonstrated (7).

Most of the COVID-19 vaccines are based on SARS-CoV-2 spike (S) glycoprotein, which is the main target for neutralizing antibodies (8), particularly the receptor-binding domain (RBD). SARS-CoV-2 S protein can have two distinct conformations, prefusion and postfusion, with the prefusion state of the S trimer being the most suitable immunogen for the development of better vaccine candidates (9). Thus, some of the most effective COVID-19 vaccines express a prefusion-stabilized S protein (Pfizer/BioNTech, Moderna and Johnson & Johnson), while others produce the non-stabilized S protein (AstraZeneca and Gamaleya).

Among SARS-CoV-2 vaccine candidates, non-replicating modified vaccinia virus Ankara (MVA) poxvirus vector have shown excellent results in preclinical trials (10–15). In fact, we have previously described that an MVA vector expressing a full-length non-stabilized SARS-CoV-2 S protein (termed MVA-CoV2-S) induced potent SARS-CoV-2-specific T-cell and humoral immune responses in C57BL/6 mice, as well as full efficacy in K18-hACE2 transgenic mice, especially when two doses of the vaccine candidate were administered (11).

Here, we have generated a novel optimized recombinant MVA virus, termed MVA-CoV2-S(3P), that expressed a full-length prefusion-stabilized S protein containing three S2 amino acid substitutions to proline to stabilize and enhance expression of the S protein (16); also, the S(3P) protein lacked the furin cleavage site to prevent S protein cleavage and fusion activation. MVA-CoV2-S(3P) expressed higher levels of the S protein in the membrane of infected cells than MVA-CoV2-S. Head-to-head comparison of the MVA-CoV2-S(3P) and MVA-CoV2-S immunogenicity and efficacy in mice, revealed that a single MVA-CoV2-S(3P) dose markedly enhanced SARS-CoV-2-specific IgG and neutralizing antibody titers against variants of concern (VoC), and controlled SARS-CoV-2 infection in K18-hACE2 transgenic mice. Our data indicated that MVA-CoV2-S(3P) is a suitable vaccine candidate to enter clinical trials, with the potential advantage in vaccination programs to limit the number of doses or use it in combination with other vaccines.

## Materials and Methods

### Ethics Statement

Female C57BL/6OlaHsd mice (6–8 weeks old) used for immunogenicity assays were purchased from Envigo Laboratories and stored in the animal facility of the Centro Nacional de Biotecnología (CNB) (Madrid, Spain). Female transgenic K18-hACE2 mice, expressing the human angiotensin converting enzyme-2 (ACE2) receptor, were obtained from the Jackson Laboratory [034860-B6.Cg-Tg(K18-ACE2)2Prlmn/J, genetic background (C57BL/6J x SJL/J)F2], and efficacy experiments were performed in the biosafety level 3 (BSL-3) facilities at the Centro de Investigación en Sanidad Animal (CISA)-Instituto Nacional de Investigaciones Agrarias (INIA-CSIC) (Valdeolmos, Madrid, Spain). The immunogenicity and efficacy animal studies were approved by the Ethical Committee of Animal Experimentation (CEEA) of the CNB (Madrid, Spain) and by the Division of Animal Protection of the Comunidad de Madrid (PROEX 49/20, 169.4/20, and 161.5/20). Animal procedures conformed with international guidelines and with Spanish law under the Royal Decree (RD 53/2013).

### Cells

DF-1 cells (a spontaneously immortalized chicken embryo fibroblast [CEF] cell line, ATCC catalog no. CRL-12203) and HeLa cells (a human epithelial cervix adenocarcinoma; ATCC catalog no. CCL-2) were grown in Dulbecco’s modified Eagle’s medium (DMEM) (Gibco-Life Technologies) supplemented with penicillin (100 U/ml; Sigma-Aldrich), streptomycin (100 μg/ml; Sigma-Aldrich), L-glutamine (2 mM; Sigma-Aldrich), nonessential amino acids (0.1 mM; Sigma-Aldrich), gentamicin (50 μg/ml; Sigma-Aldrich), amphotericin B (Fungizone, 0.5 μg/ml; Gibco-Life Technologies), and 10% heat-inactivated fetal calf serum (FCS) (Gibco-Life Technologies). Vero-E6 cells (ATCC catalog no. CRL-1586) were maintained in complete medium (DMEM supplemented with 10 mM HEPES [Gibco-Life Technologies], 1× nonessential amino acids [Gibco-Life Technologies], penicillin [100 U/ml; Sigma-Aldrich], streptomycin [100 mg/ml; Sigma-Aldrich], and 10% heat-inactivated fetal bovine serum [FBS, Gibco-Life Technologies]). Cell cultures were maintained at 37°C in a humidified incubator containing 5% CO_2_.

### Viruses

We used the attenuated MVA-WT poxvirus strain, obtained from the Chorioallantois vaccinia virus Ankara strain after 586 serial passages in CEF cells (17), and an MVA-S vaccine candidate expressing a human codon optimized full-length non-stabilized SARS-CoV-2 S protein (11). MVA-WT was used as the parental virus for the generation of the MVA-S(3P) vaccine candidate expressing a human codon optimized full-length prefusion-stabilized SARS-CoV-2 S protein. All poxviruses were grown in DF-1 cells to obtain a master seed stock (passage 2 [P2] stock) and titrated in DF-1 cells by a plaque immunostaining assay, using a rabbit polyclonal antibody against vaccinia virus (VACV) (CNB; diluted 1:1,000), followed by an anti-rabbit horseradish peroxidase (HRP)-conjugated secondary antibody (Sigma-Aldrich; diluted 1:1,000), as previously described (18). Determinations of the titers of the different viruses were performed at least two times. Furthermore, viruses grown in DF-1 cells were purified by centrifugation through two 36% (wt/vol) sucrose cushions in 10 mM Tris-HCl (pH 9). All viral stocks were free of contamination with mycoplasma (checked by PCR specific for mycoplasma), bacteria (checked by growth in LB plates without ampicillin), or fungi (checked by growth in Columbia blood agar plates; Oxoid).

SARS-CoV-2 strain MAD6 (kindly provided by José M. Honrubia and Luis Enjuanes, CNB-CSIC, Madrid, Spain) is a virus collected from a nasopharyngeal swab from a 69-year-old male COVID-19 patient from Hospital 12 de Octubre in Madrid (19). The stock virus was prepared by collecting the supernatant from Vero-E6 cells, and was isolated, plaque cloned three times, and amplified by propagation in Vero-E6 cells by inoculation at a multiplicity of infection (MOI) of 0.001 plaque-forming units (PFU)/cell (passage 2). Cell supernatants were harvested at 72 hours post-infection (hpi), cleared by centrifugation, aliquoted, and stored at −80°C. Virus infectivity titers were determined by standard plaque or median tissue culture infectious dose (TCID_50_) assays in Vero-E6 cells. Full-length virus genome was sequenced, and it was found to be identical to SARS-CoV-2 reference sequence (Wuhan-Hu-1 isolate, GenBank: MN908947), except the silent mutation C3037T, and two mutations leading to amino acid changes: C14408T (in nsp12) and A23403G (D614G in S protein).

### Construction of Plasmid Transfer Vector pCyA-S(3P)

The plasmid transfer vector pCyA-S(3P) was constructed and used for the generation of the MVA-S(3P) vaccine candidate, in which a human codon optimized full-length prefusion-stabilized SARS-CoV-2 S gene was inserted into the thymidine kinase (TK) locus of parental virus MVA-WT, under the transcriptional control of the viral synthetic early/late (sE/L) promoter. Briefly, a 3,822-kbp DNA fragment encoding a human codon optimized SARS-CoV-2 full-length prefusion-stabilized S gene (Wuhan seafood market pneumonia virus isolate Wuhan-Hu-1, GenBank accession number MN908947.3) was synthesized by GeneArt and inserted into plasmid vector pCyA (20), to generate the plasmid transfer vector pCyA-S(3P) (11,322 bp). This plasmid also contains a β-galactosidase (β-Gal) reporter gene sequence between two repetitions of the left TK-flanking arm, which allows the reporter gene to be deleted from the final recombinant virus by homologous recombination after successive passages. The encoded full-length S protein is human codon optimized and contains three amino acid mutations in the furin cleavage site (R682G, R683S, and R685S) to avoid the cleavage of the S protein in S1 and S2, and three additional proline substitutions in the S2 region that stabilize the S protein in the prefusion conformation (A942P, K986P, and V987P).

### Generation of MVA-S(3P) Vaccine Candidate

Cultured DF-1 cells (3 × 10^6^ cells) were infected with parental MVA-WT virus at a MOI of 0.05 PFU/cell and transfected 1 h later with 10 μg of DNA plasmid pCyA-S(3P), using Lipofectamine reagent according to the manufacturer’s recommendations (Invitrogen). At 48 hpi, cells were harvested, lysed by freeze–thaw cycling, sonicated, and used for recombinant virus screening. Recombinant MVA-S(3P) viruses containing the 3,822-kb SARS-CoV-2 full-length prefusion-stabilized S gene, inserted in the TK locus, and transiently coexpressing the β-Gal marker gene were selected by consecutive rounds of plaque purification in DF-1 cells stained with X-Gal (5-bromo-4-chloro-3-indolyl-β-D-galactopyranoside, 400 μg/ml, for three passages in total). In the following plaque purification steps, recombinant MVA-S(3P) viruses containing the 3,822-kb SARS-CoV-2 full-length prefusion-stabilized S gene and with the β-Gal gene deleted by homologous recombination between the left TK arm and the short left TK arm repeat flanking the marker were isolated by three additional consecutive rounds of plaque purification screening for non-staining viral foci in DF-1 cells in the presence of X-Gal (400 μg/ml). In each round of purification, the isolated plaques were expanded in DF-1 cells, and the crude viruses obtained were used for the next plaque purification round. The resulting recombinant virus MVA-S(3P) (P2 stock), was grown in DF-1 cells, purified by centrifugation through two 36% (wt/vol) sucrose cushions, and titrated by plaque immunostaining assay (18).

### Expression of SARS-CoV-2 S Protein by Western Blotting

To check the correct expression of SARS-CoV-2 prefusion-stabilized S protein by MVA-S(3P) vaccine candidate, monolayers of DF-1 or HeLa cells grown in 24-well plates were mock infected or infected at 5 PFU/cell with MVA-S(3P) (or with control viruses MVA-S, and MVA-WT). At different times (4, 7, or 24 hpi), equal amounts of cell extracts or supernatants were solubilized under reducing conditions (in the presence of 1× Laemmli plus β-mercaptoethanol), fractionated by 10% sodium dodecyl sulfate-polyacrylamide gel electrophoresis (SDS-PAGE), and analyzed by Western blotting with a rabbit polyclonal anti-SARS-CoV-2 S antibody (Genetex; diluted 1:2,000; recognizing SARS-CoV-2 S1 region) to evaluate the expression of SARS-CoV-2 S protein. For a viral loading control, we used a rabbit anti-VACV E3 (CNB; diluted 1:1,000) antibody. An anti-rabbit HRP-conjugated antibody (Sigma; diluted 1:5,000) was used as the secondary antibody. The immunocomplexes were detected with an HRP-luminol enhanced-chemiluminescence system (ECL Plus) (GE Healthcare). Band intensities were quantified using Image J software (NIH, USA).

The genetic stability of MVA-S(3P) vaccine candidate was analyzed as previously described (21). Briefly, monolayers of DF-1 cells in 35-mm flasks were infected with 0.05 PFU/cell of recombinant MVA-S(3P) (P2 stock). At 72 hpi, cells were collected by being scraped. After three freeze–thaw cycles and a brief sonication, the cellular extract was centrifuged at 1,500 rpm for 5 min, and the supernatant was used for a new round of infection of DF-1 cells at low virus multiplicity. The same procedure was repeated during 8 consecutive passages and stocks were prepared after each passage. Moreover, 13 individual plaques were picked from MVA-S(3P)-infected DF-1 cells at passage 8. Then, expression of SARS-CoV-2 S(3P) protein in DF-1 cells infected with the virus stocks at the different passages and from the 13 individual plaques at passage 8 was detected by Western blotting as described above.

### Expression of SARS-CoV-2 S Protein by Flow Cytometry

Monolayers of HeLa cells were mock infected or infected at 5 PFU/cell with MVA-WT, MVA-S, or MVA-S(3P). At 24 hpi, cells were collected by scraping and centrifuged at 2,000 rpm for 5 min, and the cellular pellets were resuspended in phosphate-buffered saline (PBS) staining solution (1× PBS, 0.5% bovine serum albumin [BSA], 1% FCS, 0.065% sodium azide, and 2 mM EDTA) and added to a 96-well plate at a rate of 200,000 cells per well. Then, Fc block (BD Pharmingen) was added for 20 min at 4°C. For the staining, mouse monoclonal antibodies against S and RBD proteins were added to the cells for 30 min at 4°C, and then anti-mouse IgG phycoerythrin (PE)-conjugated antibodies (diluted 1:100; eBioscience) were added to the cells as secondary antibodies for 20 min in the dark at 4°C. Finally, cells were fixed with 4% paraformaldehyde (PFA) for 20 min in the dark at 4°C and were acquired using a Gallios flow cytometer (Beckman Coulter). Data were analyzed using FlowJo software (version 8.5.3; Tree Star, Ashland, OR). Geometric mean fluorescence intensity (gMFI) values on the “live cells” gate were used to calculate the S and RBD scores by applying the formula: S^+^ cells × gMFI × live cells/total cells.

### Expression of SARS-CoV-2 S Protein by Confocal Immunofluorescence Microscopy

Immunofluorescence experiments were performed in HeLa cells infected at a MOI of 0.5 PFU/cell for 24 h as previously described (11, 21–23). Then, permeabilized and nonpermeabilized infected HeLa cells were stained with WGA probe conjugated to the red fluorescent dye Alexa Fluor 594 (Invitrogen; diluted 1:200) and the rabbit polyclonal antibody against SARS-CoV-2 S protein (Genetex; diluted 1:200) to label the cell membrane and the SARS-CoV-2 S protein, respectively. Anti-SARS-CoV-2 S was detected with a rabbit secondary antibody conjugated with the fluorochrome Alexa Fluor 488 (green) (Invitrogen; diluted 1:500). We used DAPI (4´,6´-diamidino-2-phenylindole; Sigma) to stain the cell nuclei. Images of sections of the cells were taken with a Leica TCS SP5 microscope.

### Peptides and Proteins

SARS-CoV-2 S peptide pools were used in the cellular immunogenicity analysis and purchased from JPT Peptide Technologies (Berlin, Germany). The S peptide pools were divided into two groups spanning the S1 and S2 regions of the S protein, with each peptide pool containing 158 (S1) or 157 peptides (S2) as consecutive 15-mers overlapping by 11 amino acids.

The SARS-CoV-2 soluble S and RBD proteins were produced in mammalian cells as previously described (11), and they were used to analyze *via* enzyme-linked immunosorbent assay (ELISA) the levels of IgG antibodies in mice serum samples. The S sequence (residues 1 to 1,208; Wuhan-Hu-1 strain, GenBank accession number MN908947.3) contained a T4 fibritin trimerization sequence, a Flag epitope, and an 8×His-tag at the C-terminus. In the S protein, the furin-recognition motif (RRAR) was replaced by the GSAS sequence, and it also contained the A942P, K986P, and V987P substitutions in the S2 portion. The S protein was purified by nickel-nitrilotriacetic acid (Ni-NTA) affinity chromatography from transfected cell supernatants and it was transferred to HEPES buffered saline (HBS), pH 7.5, during concentration or by size-exclusion chromatography (SEC).

The RBD protein with the S residues 332 to 534 was produced with an N-terminal HA-tag (YPYDVPDYA) and fused to either a T4 fibritin trimerization sequence, a Flag epitope, and an 8×His-tag (RBD-TFH). The RBD protein was purified by affinity chromatography with Ni-NTA columns (ABT) or with an anti-HA antibody bound to Sepharose.

### Immunogenicity Study Schedule in C57BL/6 Mice

To evaluate the immunogenicity of the MVA-S(3P) vaccine candidate, an MVA prime/MVA boost immunization protocol was performed in female C57BL/6 mice (6 to 8 weeks old) as previously described (11). Groups of animals (*n* = 6) received two doses of 1 × 10^7^ PFU of MVA-S(3P) or MVA-S by the intramuscular route in 100 μl of PBS (50 μl/leg) at 0 and 2 weeks. Mice primed and boosted with nonrecombinant MVA-WT were used as a control group. At 10 days after the first immunization (prime), mice were bled to obtain serum samples for titration of total IgG antibodies, IgG isotypes (IgG1, IgG2c, and IgG3), and neutralizing antibodies against SARS-CoV-2. Then, at 10 days after the last immunization (boost), mice were sacrificed using carbon dioxide (CO_2_). Blood from each individual mouse was collected by cardiac puncture and processed to obtain serum samples to analyze SARS-CoV-2-specific humoral immune responses; the spleens of each group were pooled and processed to measure the adaptive T-cell immune responses to the SARS-CoV-2 S antigen by Enzyme-Linked ImmunoSpot (ELISpot) and intracellular cytokine staining (ICS) assays. No adverse effects were detected in immunized mice.

### ELISpot Assay

The ELISpot assay was used to detect SARS-CoV-2 S-specific IFNγ-secreting cells. First, 96-well nitrocellulose-bottom plates (Millipore) were covered with 75 μl/well of a solution of the rat anti-mouse IFNγ monoclonal antibody (Pharmingen) at a concentration of 6 μg/ml in PBS. After incubating overnight at room temperature, the wells were washed three times with RPMI medium and blocked with RPMI-10% FCS for at least 1 h at 37°C in 5% CO_2_ atmosphere. After spleen processing, 10^6^ splenocytes per condition were re-stimulated with 1 μg/ml of the SARS-CoV-2 S1 or S2 peptide pools or with RPMI-10% FCS. The plates were incubated with the peptides for 48 h at 37°C in 5% CO_2_ atmosphere, washed 5 times with PBS-Tween 20, and incubated with 2 μg/ml of biotinylated rat anti-mouse IFNγ monoclonal antibody XMG1.2 (Pharmingen) diluted in PBS-Tween 20, for 2 h at room temperature. The plates were then washed 5 times with PBS-Tween 20 and a 1:800 dilution of HRP-conjugated streptavidin (0.5 mg/ml; Sigma-Aldrich) was added. After 1 h at room temperature, they were washed 3 times with PBS-Tween 20 and 2 times with PBS, and finally, 1 μg/ml of the DAB substrate (Sigma-Aldrich) was resuspended in 50 mM Tris-Cl, pH 7.5, and 0.015% H_2_O_2_ was added to develop the plates. The reaction was stopped by plate washing with abundant water, and once it was dry, the spots were counted using the ELISpot Reader System-ELR02-plate reader (AID Autoimmun Diagnostika GmbH) with the aid of AID ELISpot reader system software (Vitro).

### ICS Assay

The magnitude, breadth, polyfunctionality, and memory phenotype of adaptive SARS-CoV-2 S-specific CD4^+^ and CD8^+^ T cells expressing CD107a, and/or IFNγ, and/or TNFα, and/or IL-2 were analyzed by an ICS assay as previously described (11) in splenocytes stimulated with SARS-CoV-2 S1 and S2 peptide pools (JPT Peptide Technologies). Cells were acquired with a Gallios flow cytometer (Beckman Coulter), and analyses of the data were performed with the FlowJo software version 10.4.2 (Tree Star), as previously described (11).

### ELISA

The titers of binding IgG, IgG1, IgG2c and IgG3 anti-S and -RBD antibodies in individual or pooled sera from immunized C57BL/6 or K18-hACE2 mice were measured by ELISA as previously described (11). Total binding IgG titers were measured as the last serum dilution that gave an absorbance value at 450 nm at least three times higher the absorbance of a naive serum.

### SARS-CoV-2 Neutralization

Capacity of the sera obtained from C57BL/6 or K18-hACE2 mice immunized with MVA-S and MVA-S(3P) to neutralize live SARS-CoV-2 virus was determined using a microneutralization test (MNT) assay in a BSL-3 laboratory at the CNB-CSIC. Serially twofold diluted mouse serum samples in DMEM-2% FBS medium were incubated at a 1:1 ratio with 100 TCID_50_ of the SARS-CoV-2 MAD6 isolate in 96-well tissue culture plates for 1 h at 37°C. Then, mixtures of serum samples and SARS-CoV-2 virus were added in duplicate to Vero-E6 cell monolayers seeded in 96-well plates at 30,000 cells/well, and the plates were incubated at 37°C, in a 5% CO_2_ incubator for 3 days. Then, cells were fixed with 10% formaldehyde for 1 h and stained with crystal violet. When plates were dried, crystal violet was diluted in H_2_O-10% SDS and optical density was measured in a luminometer at 570 nm. Titers of neutralizing antibodies were determined as the reciprocal highest serum dilution that resulted in a 50% inhibition of cell death (neutralizing titer 50 [NT_50_]) following a methodology previously described (24). A WHO International Standard containing pooled plasma obtained from eleven individuals recovered from SARS-CoV-2 infection (NIBSC code: 20/136) was used for the calibration and harmonization of the MNT assay.

### Neutralization of SARS-CoV-2 Variants of Concern

Capacity of serum samples to neutralize different SARS-CoV-2 VoC was tested by using SARS-CoV-2 pseudotyped Vesicular Stomatitis Virus (VSV) expressing S protein. SARS-CoV-2 S-protein pseudotyped rVSV-luc recombinant viruses (PSV) were produced as described elsewhere (25). SARS-CoV-2 S variants used were S_614D, S_614G, alpha (B.1.1.7), beta (B.1.351), gamma (P.1) and delta (B.1.617.2). SARS-CoV-2 S mutant D614G was generated by site-directed mutagenesis (Q5 Site Directed Mutagenesis Kit; New England Biolabs) following the manufacturer’s instructions and using as an input DNA a pcDNA3.1 expression vector encoding SARS-CoV-2 S_614D (11). SARS-CoV-2 VoC alpha (B.1.1.7; GISAID: EPI_ISL_608430), beta (B.1.351; GISAID: EPI_ISL_712096), gamma (P.1; GISAID: EPI_ISL_833140), and delta (B.1.617.2; GISAID: EPI_ISL_1970335) were optimized, synthesized, and cloned into pcDNA3.1 by GeneArt (Thermo Fisher Scientific, GeneArt GmbH, Regensburg, Germany).

The neutralization activity of serum samples was tested by triplicates at several twofold dilutions. For neutralization experiments, viruses-containing transfection supernatants were normalized for infectivity to a MOI of 0.5–1 and incubated with the dilutions of serum samples at 37°C for 1 h in 96-well plates. After the incubation time, 2 × 10^4^ Vero-E6 cells were seeded onto the virus–serum mixture and incubated at 37°C for 24 h. Cells were then lysed and assayed for luciferase expression, and NT_50_ titers were calculated using a nonlinear regression model fit with settings for log agonist/inhibitor versus normalized response curves, in GraphPad Prism v8.

### Efficacy Study Schedule in K18-hACE2 Transgenic Mice

Female and male K18-hACE2 mice (9 weeks old at the beginning of the study) immunized with one dose of MVA-S(3P) were used to evaluate its efficacy of the MVA-S(3P) vaccine candidate. Groups of animals (*n* = 11) received one dose of 1 × 10^7^ PFU of MVA-S(3P) by intramuscular route in 100 μl of PBS (50 μl/leg). Mice immunized with MVA-S, or with nonrecombinant MVA-WT were used as control groups. At week 4, all mice were challenged with a lethal dose (1 × 10^5^ PFU) of SARS-CoV-2 (MAD6 strain) by intranasal route in 50 μl of PBS after being anesthetized in an isoflurane chamber. Mice were monitored for body weight change and mortality for 15 days postchallenge. Animals with more than a 25% of weight loss were euthanized by cervical dislocation, using forceps. At 4 days postchallenge, six mice per group were euthanized, and lung and serum samples were collected. The entire left lung lobe was removed from each mouse and immersion-fixed in zinc formalin (Sigma-Aldrich) for 48 h. After the fixation period, samples were routinely processed and embedded in paraffin for subsequent histopathological evaluations. Right lung lobes were divided longitudinally into two, with one part placed in RNALater stabilization reagent (Sigma-Aldrich) and stored at −80°C until RNA extraction, and the other part stored at −80°C until analysis of virus yields. Blood was collected by submandibular bleeding, maintained at 37°C for 1 h, kept at 4°C overnight, and centrifuged at 3,600 rpm for 20 min at 4°C to obtain the serum samples, which was then inactivated at 56°C for 30 min and kept at −20°C until use.

### Analysis of SARS-CoV-2 RNA by Quantitative RT-PCR

Lungs from K18-hACE2 mice were harvested at 4 days postchallenge and stored in RNALater (Sigma-Aldrich) at −80°C until homogenized with a gentleMACS dissociator (Miltenyi Biotec) in 2 ml of RLT buffer (Qiagen) plus β-mercaptoethanol (Sigma-Aldrich) and aliquoted. Then, 600 μl of homogenized lung tissue was used to isolate total RNA using the RNeasy minikit (Qiagen), according to the manufacturer’s specifications. First-strand cDNA synthesis and subsequent real-time PCR were performed in one step using NZYSpeedy One-step RT-qPCR Master Mix (NZYTech), according to the manufacturer’s specifications using ROX as reference dye. SARS-CoV-2 viral RNA content was determined using previously validated set of primers and probes specific for the SARS-CoV-2 subgenomic RNA for the protein E, the genomic virus RNA dependent RNA polymerase (RdRp) gene and the cellular 28S rRNA for normalization (26). Data were acquired with a 7500 real-time PCR system (Applied Biosystems) and analyzed with 7500 software v2.0.6. Relative RNA arbitrary units (A.U.) were quantified relative to a negative group (uninfected mice) and were performed using the 2^−ΔΔCt^ method. All samples were tested in duplicate.

### Analysis of SARS-CoV-2 Virus Yields by Plaque Assay

Lungs from K18-hACE2 mice were harvested at 4 days postchallenge, weighted, and stored directly at −80°C until homogenized with a gentleMACS dissociator (Miltenyi Biotec) in 2 ml of PBS buffer and aliquoted. Then, undiluted and serial tenfold dilutions of homogenized lung tissue were added in triplicate to Vero-E6 cell monolayers seeded in 12-well plates at 5 × 10^5^ cells/well and after 1 h of adsorption the inoculum was removed and plates were incubated at 37°C, 5% CO_2_ in 2:1 DMEM 2X-4% FBS:2% Agar. After 4 days, cells were fixed for 1 h with 10% formaldehyde (Sigma-Aldrich), then the agarose was removed and plaques were visualized by adding 0.5% crystal violet (Sigma-Aldrich). SARS-CoV-2 titers were determined in PFUs per gram of lung tissue.

### Lung Cytokine Profile Analysis by RT-qPCR

Reverse transcription of 4,000 ng of RNA isolated as described above from lung homogenates of K18-hACE2 mice was performed with the QuantiNova reverse transcription kit (Qiagen), according to the manufacturer’s recommendations. RT-qPCR was performed with a 7500 real-time PCR system (Applied Biosystems) using Power SYBR green PCR Master Mix (Applied Biosystems), as previously described (27). The mRNA expression levels of IP-10, IL-12b, CCL11, and IFN-γ genes were analyzed by real-time PCR with specific oligonucleotides (sequences are available upon request). Specific gene expression was expressed relative to the expression of the cellular 28S ribosomal RNA gene in fold change units using the 2^−ΔΔCt^ method. All samples were tested in triplicate.

### Lung Histopathology

The entire left lung lobe was removed from each K18-hACE2 mouse and immersion-fixed in zinc formalin (Sigma-Aldrich) for 48 h. After the fixation period, samples were routinely processed and embedded in paraffin blocks that were then sectioned at 4 µm thickness on a microtome, mounted onto glass slides, and routinely stained with haematoxylin and eosin (H&E). Lung sections were microscopically evaluated using an Olympus BX43 microscope by a single veterinary pathologist who was blinded to the identity and group of individual mice. To assess the character and severity of histopathological lesions, lung inflammation scoring parameters based on previous reports on SARS-CoV-2 infection in mouse models were used (28). The histopathological parameters evaluated were as follows: capillary endothelial cell activation; alveolar hemorrhages; alveolar edema; perivascular edema; alveolar septal thickening (interstitial pneumonia); alveolar damage and hyaline membranes in alveoli; inflammatory cell infiltration in alveoli; bronchi/bronchioles with epithelial necrosis; detached epithelium or inflammatory cells in the lumen (bronchitis/bronchiolitis); peribronchial/peribronchiolar and perivascular mononuclear infiltrates; pneumocytes hyperplasia; cytopathic effect or syncytia; squamous metaplasia; uniform interstitial fibrosis; organized fibrotic tissue around the bronchi/bronchioles or intrabronchiolar (bronchiolitis obliterans); and pleural thickening. The histopathological parameters were graded following a semi-quantitative scoring system as follows: (0) no lesion; (1) minimal lesion; (2) mild lesion; (3) moderate lesion and (4) severe lesion. The cumulative scores of histopathological lesions provided the total score per animal. In each experimental group, the individual scores were used to calculate the group average. In addition, H&E-stained sections were visually scored 0–6 based on the percentage of lung area affected by inflammatory lesions as follows: 0% of the lung injured (score 0); <5% (score 1); 6%–10% (score 2); 11%–20% (score 3); 21%–30% (score 4); 31%–40% (score 5); >40% (score 6). In each experimental group, the individual scores were used to calculate the group average.

### Statistical Procedures

For statistical analysis, one-way ANOVA of transform data followed by post-hoc Student’s *t*-test comparisons was used to establish the differences between two groups. Statistical analysis of the ICS assay results were realized as previously described (29), using an approach that corrects measurements for background response, calculating confidence intervals and *p*-values. The statistical significances are indicated as follows: *p < 0.05; **p < 0.005; ***p < 0.001.

## Results

### MVA-CoV2-S(3P) Vaccine Candidate Expressed Higher Amounts of the SARS-CoV-2 S Protein than MVA-CoV2-S on Infected Cell Membranes

**Figure 1:**
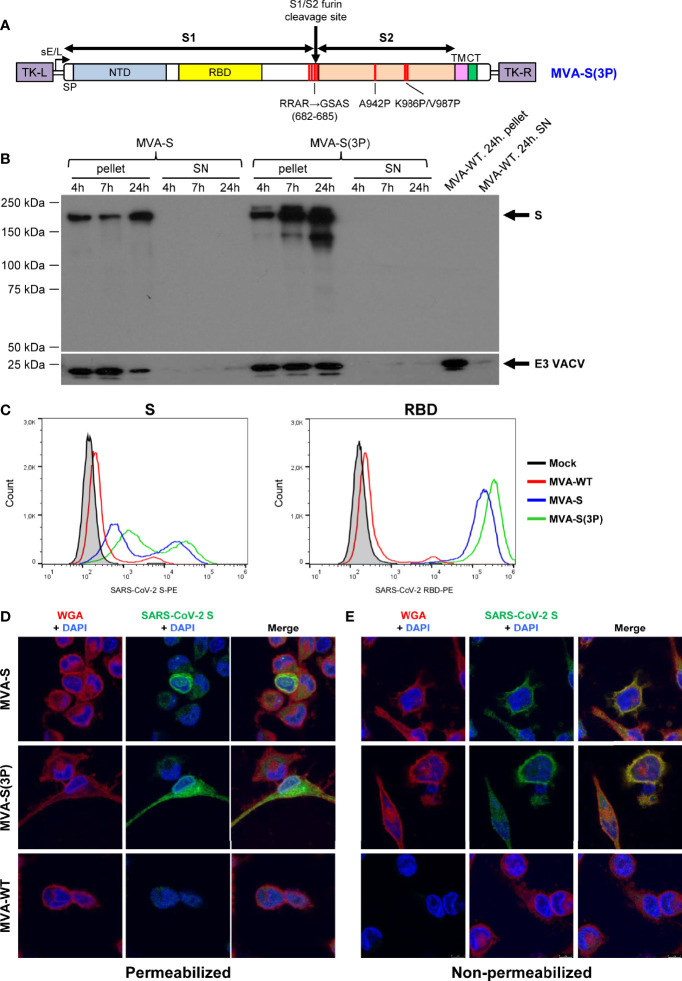
Design, generation, and *in vitro* characterization of MVA-S(3P) vaccine candidate. **(A)** Scheme of the prefusion-stabilized full-length S protein inserted in the MVA genome to generate the MVA-S(3P) vaccine candidate. S1 and S2 regions are indicated, together with the amino acid mutations in the furin cleavage site and changes to prolines in the S2 region. The SARS-CoV-2 S gene is inserted within the TK locus of MVA-WT virus and is driven by the sE/L virus promoter. NTD: N-terminal domain; RBD: receptor binding domain; TM: transmembrane; CT: cytoplasmic tail; TK-L: TK left; TK-R: TK right. **(B)** Expression of SARS-CoV-2 S protein by MVA-S and MVA-S(3P) vaccine candidates. Western blotting of MVA-infected (5 PFU/cell) DF-1 cell extracts (pellet) and corresponding supernatants (SN) at 4, 7, and 24 hpi. Rabbit polyclonal anti-S and anti-VACV E3 antibodies were used for protein identification on 7% SDS-PAGE under reducing conditions. Size (in kilodaltons [kDa]) and migration of molecular weight markers are indicated. **(C)** Flow cytometry analysis of S expression levels in cells infected with MVA-S(3P). HeLa cells were mock infected or infected at 5 PFU/cell with MVA-S, MVA-S(3P), and MVA-WT, and at 24 hpi, cells were collected and analyzed by flow cytometry using mouse polyclonal antibodies against S and RBD proteins, followed by an anti-mouse PE-conjugated antibody. Cell surface expression of S protein is represented in the histogram plots. **(D, E)** Subcellular distribution of SARS-CoV-2 S protein in cells infected with MVA-S and MVA-S(3P). Confocal immunofluorescence microscopy of infected and permeabilized **(D)** or nonpermeabilized **(E)** HeLa cells. Cells were infected with 0.5 PFU/cell with the indicated viruses, fixed at 24 hpi, permeabilized or nonpermeabilized, and stained with Alexa Fluor 594-conjugated WGA probe (red) and with a rabbit polyclonal anti-S antibody further detected with a rabbit Alexa Fluor 488-conjugated antibody (green). Cell nuclei were stained using DAPI (blue).

The generation of an MVA vector expressing a full-length non-stabilized SARS-CoV-2 S protein (MVA-CoV2-S or MVA-S in the abbreviated form) and its immunogenic and efficacy in mice was previously described (11). However, it has been reported that stabilization of SARS-CoV-2 S protein in a prefusion state by substitution of two or more amino acids to prolines in the S2 region of the S sequence and mutation of the furin cleavage site can increase S expression and improve immunogenicity and efficacy of vaccines against SARS-CoV-2 (8, 9, 16). Thus, to enhance the immunogenicity and efficacy of our MVA-S vaccine candidate, we generated a novel MVA-CoV2-S(3P) vector [termed MVA-S(3P) in the abbreviated form] that expressed a human codon-optimized full-length prefusion-stabilized SARS-CoV-2 S protein. To increase S protein expression and to stabilize its prefusion form, three amino acid substitutions to prolines were introduced in the S2 region (A942P, K986P, and V987P), and the furin cleavage site removed to avoid the processing of the S into the S1 and S2 regions and fusion activation (**Figure 1A**).

The correct generation and purity of MVA-S(3P) was confirmed by PCR and DNA sequencing of the whole viral DNA genome (data not shown). Analysis by Western blotting of cell extracts and supernatants obtained at different times post-infection from permissive chicken DF-1 cells infected with MVA-S(3P) showed only cell-associated S protein that migrated with a size about 180 kDa, similar to the native S (**Figure 1B**), and as expected for a full-length glycosylated S protein containing the transmembrane domain. Other lower size bands detected were probably the result of some proteolytic processing, furin independent. The S protein was expressed as early as 4 hpi and increased with time (**Figure 1B**).

MVA-S(3P) stability was determined by analysis of SARS-CoV-2 S protein expression during successive virus passages in permissive DF-1 cell cultures at low MOI (0.05 PFU/cell). The Western blot results showed that MVA-S(3P) efficiently expressed the S protein during 8 successive passages, indicating virus genetic stability in cell cultures (**Supplementary Figure S1A**). Moreover, all individual plaques isolated from MVA-S(3P) at passage 8 showed stable S protein expression (**Supplementary Figure S1B**). The stability of MVA-S from individual virus plaques was previously described (11).

In addition, we compared S protein expression from non-permissive human HeLa cells infected with either MVA-S(3P) or MVA-S at different times post-infection. The Western blot results showed a major 180-kDa protein product and some differences in cleavage products between cells infected with MVA vectors expressing unmodified and modified S protein. From the band intensities, normalized to VACV E3 protein, and quantified by Image J software, it was estimated that expression of SARS-CoV-2 S protein was about twofold higher for cells infected with MVA-S(3P) over MVA-S (**Supplementary Figure S1C**).

Remarkably, analysis of S protein expression by flow cytometry in HeLa cells at 24 hpi confirmed the higher expression levels of S protein on the membrane of cells infected with MVA-S(3P) than with MVA-S (**Figure 1C**). A quantitation of S expression revealed a 2- and 1.5-fold increase in S and RBD scores, respectively, of MVA-S(3P) over MVA-S (data not shown).

Confocal immunofluorescence microscopy localized the protein in the cytoplasm of permeabilized infected HeLa cells (**Figure 1D**), whereas in non-permeabilized infected cells, the S protein colocalized with the wheat germ agglutinin (WGA) cell membrane marker (**Figure 1E**), confirming that the SARS-CoV-2 S protein expressed by MVA-S(3P) is a membrane-bound protein.

### One Dose of MVA-S(3P) Elicited Higher Titers of SARS-CoV-2-Specific IgGs and Neutralizing Antibodies Than MVA-S in Immunized C57BL/6 Mice

**Figure 2:**
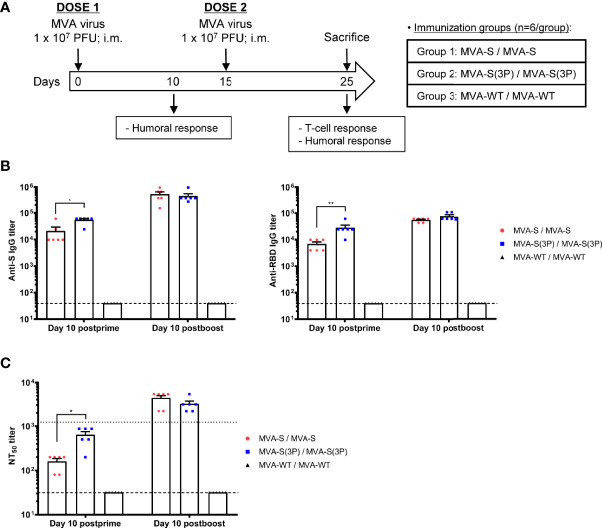
Immunization schedule in C57BL/6 mice vaccinated with MVA-S and MVA-S(3P) vaccine candidates and humoral responses induced. **(A)** Immunization schedule in C57BL/6 mice. Six C57BL/6 mice per group were inoculated at days 0 and 15 (prime/boost) with vaccine candidates MVA-S or MVA-S(3P), as indicated. At day 10, post-prime mice were bled to obtain serum samples. Mice were sacrificed 10 days after the boost (day 25) to analyze the adaptive SARS-CoV-2-specific T-cell and humoral immune responses. PFU: Plaque-forming units; i.m.: intramuscular. **(B)** Titers of IgG antibodies specific for the S (left) and RBD (right) proteins (Wuhan strain) at 10 days post-prime or 10 days post-boost. Determined by ELISA in individual mouse serum samples collected at 10 days post-prime and at 10 days post-boost from immunized C57BL/6 mice. Mean values and SEM are represented. Dashed line represents the limit of detection. **(C)** SARS-CoV-2 neutralizing antibody titers. NT_50_ titers were evaluated in individual mouse serum samples collected at 10 days post-prime and at 10 days post-boost from immunized C57BL/6 mice, using a live virus microneutralization assay. Mean NT_50_ values and SEM are represented. Upper dotted line represents the levels obtained with the NIBSC 20/136 international standard plasma (containing pooled plasma obtained from 11 individuals recovered from SARS-CoV-2 infection). Bottom dotted line represented the limit of detection. Student’s *t*-test: *p < 0.05; **p < 0.005.

Next, we evaluated the *in vivo* immunogenicity induced by MVA-S(3P) and compared it to MVA-S. Groups of C57BL/6 mice (*n* = 6) were immunized twice with MVA-S(3P) or MVA-S intramuscularly at weeks 0 and 2 (**Figure 2A**), and 10 days after the first (priming) and second (booster) dose, SARS-CoV-2-specific humoral immune responses were analyzed (**Figure 2A**).

Serum samples collected after the first MVA vaccine dose showed significantly higher titers of IgG antibodies against S and RBD proteins in mice immunized with MVA-S(3P) than with MVA-S (**Figure 2B**). After the second dose, the levels of IgG antibodies increased, and both vaccine candidates induced similar titers (**Figure 2B**). Furthermore, in both vaccinated groups, S- and RBD-specific IgG1, IgG2c, and IgG3 antibodies were induced with IgG2c > IgG1 > IgG3 and IgG2c/IgG1 ratios above 1, indicative of a Th1-like protective immune response (**Table 1**) (30).

**Table 1:** Isotype analysis of anti-S and anti-RBD IgG antibodies in immunized C57BL/6 and transgenic K18-hACE2 mice.

Time points analyzed	IgG1, IgG2c, and IgG3 titers and IgG2c/IgG1 ratio in prime/boost immunized C57BL/6 mice^a^															
	MVA-S/MVA-S								MVA-S(3P)/MVA-S(3P)							
	S				RBD				S				RBD			
	IgG1	IgG2c	IgG3	IgG2c/IgG1	IgG1	IgG2c	IgG3	IgG2c/IgG1	IgG1	IgG2c	IgG3	IgG2c/IgG1	IgG1	IgG2c	IgG3	IgG2c/IgG1
10 days postprime	625	3,906	250	6.25	250	3,906	250	15.62	1,562	9,765	625	6.25	625	9,765	1,562	15.62
10 days postboost	17,089	61,035	1,562	3.57	3,906	24,414	625	6.25	3,906	61,035	1,562	15.63	1,562	24,414	1,562	15.63
	**IgG1, IgG2c, and IgG3 titers and IgG2c/IgG1 ratio in transgenic K18-hACE2 mice**^a^															
	**MVA-S/SARS-CoV-2**								**MVA-S(3P)/SARS-CoV-2**							
	**S**				**RBD**				**S**				**RBD**			
	**IgG1**	**IgG2c**	**IgG3**	**IgG2c/IgG1**	**IgG1**	**IgG2c**	**IgG3**	**IgG2c/IgG1**	**IgG1**	**IgG2c**	**IgG3**	**IgG2c/IgG1**	**IgG1**	**IgG2c**	**IgG3**	**IgG2c/IgG1**
14 days postprime (prechallenge)	1,562	9,765	250	6.25	625	3,906	250	6.25	9,765	61,035	3,906	6.25	9,765	24,414	1,562	2.50
Day 4 postchallenge	625	1,562	250	2.50	250	625	250	2.50	3,906	9,765	625	2.50	1,562	9,765	625	6.25
Day 15 postchallenge	24,414	381,467	1,562	15.62	9,765	381,467	1,562	39.06	9,765	152,587	1,562	15.63	9,765	61,035	250	6.25

a Mean titer of IgG1, IgG2c, and IgG3 isotype antibodies against S and RBD proteins from duplicates of pooled sera samples obtained from the different immunization regimens studied are represented, including the IgG2c/IgG1 ratio.

Similarly, the SARS-CoV-2 antibody neutralization titers induced by a single MVA-S(3P) immunization dose in mice were significantly higher than those with MVA-S (**Figure 2C**). Nonetheless, the neutralization titers in both vaccinated groups were similar after the second vaccine dose (**Figure 2C**). Moreover, neutralization titers induced after two doses were higher than those of an anti-SARS-CoV-2 human immunoglobulin WHO international standard (NIBSC 20/136) (**Figure 2C**). Similar results were obtained using retrovirus-based pseudoparticles expressing the S protein (data not shown)

### MVA-S(3P) Induced Similar Strong, Polyfunctional and Effector Memory SARS-CoV-2 S-Specific CD4^+^ and CD8^+^ T-Cell Immune Responses Compared to MVA-S in C57BL/6 Mice Immunized With Two Doses

S-specific T-cell immune responses elicited by MVA-S(3P) were next analyzed, and compared to MVA-S, in immunized C57BL/6 mice at 10 days post-boost, as described in **Figure 2A**.

**Figure 3:**
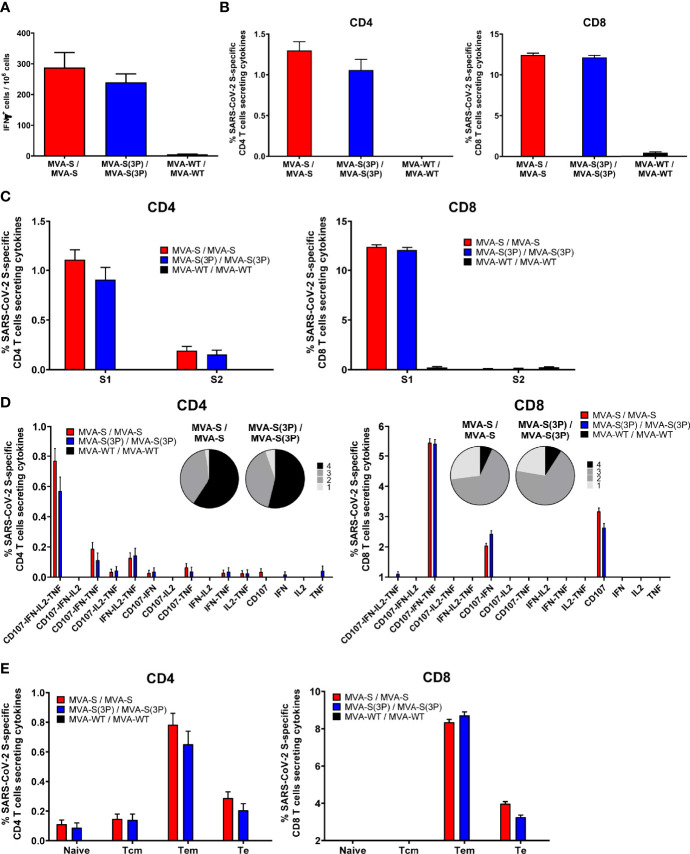
SARS-CoV-2 S-specific T-cell immune responses elicited in C57BL/6 immunized mice. **(A)** Magnitude of SARS-CoV-2 S-specific cell responses. Cells secreting IFN-γ per million of splenocytes and directed against S1 + S2 peptide pools in C57BL/6 immunized mice were evaluated at 10 days post-boost by an ELISpot assay from a pool of splenocytes derived from 6 immunized mice per group. Mean values and SEM of triplicate samples. **(B)** Magnitude of S-specific CD4^+^ and CD8^+^ T-cell immune responses evaluated at 10 days post-boost. Percentages of CD4^+^ or CD8^+^ T cells expressing CD107a and/or producing IFN-γ and/or TNF-α and/or IL-2 against a mixture of S1 and S2 peptide pools in immunized mice. Cell percentages were determined by ICS from splenocyte pools. **(C)** S-specific T-cell immune responses against S1 and S2 regions. Percentages of S1- or S2-specific CD4^+^ and CD8^+^ T cells determined as in panel **(B)**. **(D)** Polyfunctional profiles (based on expression of selected markers CD107a, IFN-γ, TNF-α and IL-2) of total S-specific CD4^+^ or CD8^+^ T-cell immune responses directed against a mixture of S1 and S2 peptide pools. The pie charts summarize the percentage of S-specific T cells exhibiting 1, 2, 3, or 4 markers. **(E)** Percentages of Naïve (CD127^-^/CD62L^-^), T central memory (Tcm, CD127^+^/CD62L^+^), T effector memory (Tem, CD127^+^/CD62L⁻), and T effector (Te, CD127⁻/CD62L⁻) CD4^+^ and CD8^+^ T cells specific for S1 and S2 peptide pools, and expressing any of the markers CD107a, IFN-γ, TNF-α, and IL-2.

Mice vaccinated with two doses of MVA-S(3P) or MVA-S elicited similar high levels of IFNγ-secreting cells, reactive to a mixture of SARS-CoV-2 S1+S2 peptide pools, as revealed by ELISpot (**Figure 3A**). Potent and similar S-specific CD4^+^ and CD8^+^ T-cells expressing CD107a, and secreting IFNγ, TNFα, and/or IL-2 were induced by both vaccine candidates, with a higher overall response mainly mediated by the CD8^+^ T-cell compartment and a CD4^+^ Th1-skewed profile, as measured by intracellular cytokine staining (ICS) (**Figure 3B**). Furthermore, S-specific CD4^+^ and CD8^+^ T-cell responses were directed mainly against the S1 peptide pool in both vaccinated groups (**Figure 3C**), with also S2-specific CD4^+^ T cells being detected (**Figure 3C**, left). Moreover, MVA-S(3P) and MVA-S triggered similar highly polyfunctional CD4^+^ and CD8^+^ T cells (**Figure 3D**), with approximately 80% and 55% of S-specific CD4^+^ and CD8^+^ T cells, respectively, exhibiting three or four functions (**Figure 3D**, pie charts). In particular, CD4^+^ T cells expressing CD107a-IFNγ-TNFα-IL-2 and CD8^+^ T cells expressing CD107a-IFNγ-TNFα were the most abundant CD4^+^ and CD8^+^ T-cell populations in both vaccinated groups (**Figure 3D**, bars). In addition, S-specific CD4^+^ and CD8^+^ T cells induced by MVA-S(3P) and MVA-S-vaccinated groups displayed mainly a T effector memory phenotype, with similar levels in both experimental groups (**Figure 3E**).

### One Single Dose of MVA-S(3P) Prevented Morbidity and Mortality in SARS-CoV-2-Challenged K18-hACE2 Transgenic Mice, Reducing SARS-CoV-2 Virus Replication, Lung Pathology, and Levels of Pro-inflammatory Cytokines

**Figure 4:**
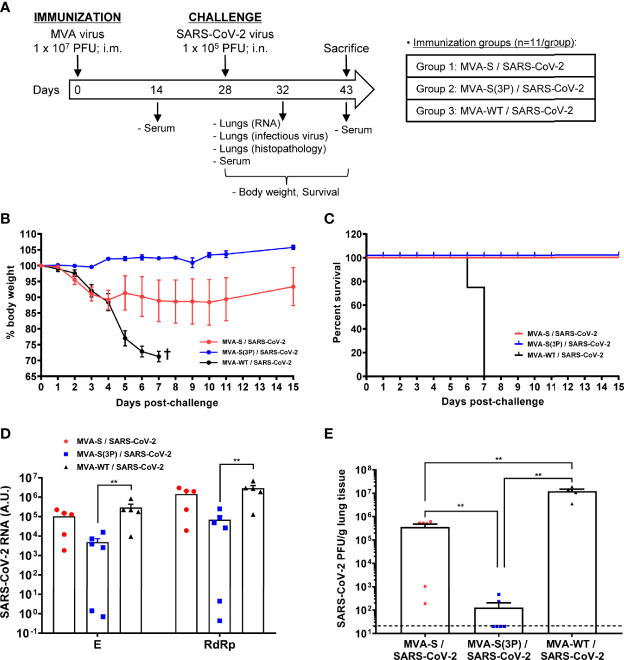
One dose of MVA-S(3P) protects transgenic K18-hACE2 mice from SARS-CoV-2 infection. **(A)** Efficacy schedule. Male and female K18-hACE2 transgenic mice (*n* = 11 per group) were immunized by the intramuscular (i.m.) route with one dose of 1 × 10^7^ PFU of MVA-S(3P) or MVA-S as indicated. At week 4 (day 28), mice were challenged intranasally (i.n.) with 1 × 10^5^ PFU of SARS-CoV-2 (MAD6 isolate). MVA-WT-inoculated mice were also i.n. challenged with SARS-CoV-2 and used as a control. At day 4 postchallenge, 6 mice per group were sacrificed and lungs and serum samples were collected as indicated. Serum was also collected at 14 days after immunization and in mice alive at 15 days postchallenge (groups 1 and 2). **(B, C)** The challenged mice were monitored for change of body weights **(B)** and mortality **(C)** for 15 days. †: mice were euthanized due to loss of more than 25% of initial body weight. **(D)** Virus replication in lung samples. Subgenomic **(E)** and genomic (RdRp) SARS-CoV-2 RNA detected by RT-qPCR at 4 days after virus infection (*n* = 6). Mean RNA levels (in arbitrary units [A.U.]) and SEM from duplicates of each lung sample; relative values are referred to uninfected mice. **(E)** SARS-CoV-2 infectious virus in lung samples. Mean (PFU/g of lung tissue) and SEM from triplicates of each lung sample. Student’s *t*-test: **p < 0.005.

As we observed that one single dose of MVA-S(3P) was more immunogenic than one dose of MVA-S in C57BL/6 mice, we next evaluated the efficacy triggered by one dose of both vaccine candidates in transgenic K18-hACE2 mice, susceptible to SARS-CoV-2 infection (31, 32). K18-hACE2 mice (*n* = 11/group) were intramuscularly immunized with one dose of MVA-S(3P) or MVA-S, and challenged 4 weeks later with a lethal dose of SARS-CoV-2 (MAD6 isolate containing D614G mutation) by intranasal route (**Figure 4A**). Challenged mice previously inoculated with one dose of MVA-WT were used as a control group.

To evaluate the vaccine efficacy, mice were monitored for changes in body weight and mortality for 15 days after the challenge. All K18-hACE2 mice immunized with one dose of MVA-S(3P) and challenged with SARS-CoV-2 did not lose body weight (**Figure 4B**) and survived (**Figure 4C**), whereas mice immunized with one dose of MVA-S lost body weight during the first 4 days postchallenge (**Figure 4B**), but recovered and survived (**Figure 4C**). On the other hand, mice inoculated with MVA-WT and challenged with SARS-CoV-2 lost body weight (more than 25%) (**Figure 4B**) and all died at 6–7 days postchallenge (**Figure 4C**).

To determine the effect of vaccination in SARS-CoV-2 virus replication, six mice per group were sacrificed at day 4 after SARS-CoV-2 virus challenge, lungs were collected and processed, and the presence of SARS-CoV-2 subgenomic E and genomic RdRp RNA (**Figure 4D**) as well as of live infectious virus (**Figure 4E**) was analyzed. One dose of MVA-S(3P) was more effective than MVA-S to prevent SARS-CoV-2 replication, reducing significantly subgenomic and genomic SARS-CoV-2 RNA levels (**Figure 4D**) in comparison to MVA-WT control infected mice, as well as the infectious virus yields with respect to the MVA-S vaccinated mice and MVA-WT control infected mice (**Figure 4E**).

**Figure 5:**
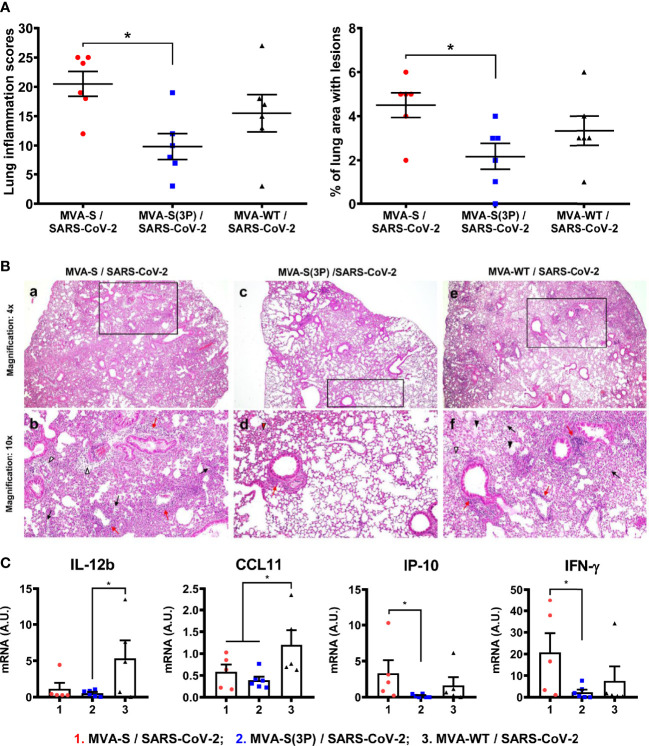
One dose of MVA-S(3P) reduced SARS-CoV-2 lung pathology and diminished levels of pro-inflammatory cytokines in K18-hACE2 transgenic mice. **(A)** Lung inflammation scores (left) and percentage of lung area with lesions (right) examined in lung samples (*n* = 6) at 4 days postchallenge. Mean and SEM of cumulative histopathological lesion scores (left) and percentage of lung area affected by inflammatory lesions (right). **(B)** Representative lung histopathological sections (H&E staining) from K18-hACE2 transgenic mice euthanized at day 4 postchallenge. A general view of the lung area (magnification: 4×) along with histopathological details from selected lung areas (black boxes) have been displayed (magnification: 10×). Mice vaccinated with one dose of MVA-S and MVA-WT (a,e) displayed more extensive and severe lung inflammatory lesions compared to mice immunized with one dose of MVA-S(3P) (c). In mice immunized with MVA-S and MVA-WT (b,f), lung inflammatory lesions were characterized by the presence of moderate diffuse thickening of the alveolar septae, perivascular edema (white arrowheads), moderate presence of mononuclear cell infiltrates within alveolar spaces (black arrows), hyaline membranes and cell debris within alveoli (black arrowheads), as well as the presence of large multifocal perivascular and peribronchiolar mononuclear infiltrates (red arrows). However, in mice vaccinated with one dose of MVA-S(3P) (d), lesions were less extensive and less severe, highlighting the presence of focal thickening of the alveolar septae (red arrowhead), occasional presence of inflammatory cells within the alveoli, and small focal perivascular and peribronchial mononuclear infiltrates (red arrows). **(C)** Identification of proinflammatory cytokines. mRNA levels detected by RT-qPCR in lungs (*n* = 6) obtained at 4 days postchallenge. Mean RNA levels (in A.U.) and SEM from duplicates of each lung sample; relative values are referred to uninfected mice. Student’s *t*-test: *p < 0.05.

Histopathological evaluation of lungs at 4 days postchallenge (*n* = 6/group) showed that mice vaccinated with one dose of MVA-S(3P) exhibited lower lung inflammation scores (**Figure 5A**, left panel) and lower percentages of lung area with lesions (**Figure 5A**, right panel) than control MVA-WT mice, and significantly lower lesions and lung affected area than mice immunized with one dose of MVA-S (**Figure 5A**). Representative images of lung sections are included in **Figure 5B**; mice vaccinated with one dose of MVA-S(3P) only displayed focal thickening of the alveolar septae, and occasional presence of inflammatory cells within the alveoli. However, mice immunized with one dose of MVA-S or with control MVA-WT showed more severe diffuse thickening of the alveolar septae, higher presence of mononuclear cell infiltrates within alveolar spaces, as well as the presence of larger multifocal perivascular and peribronchiolar mononuclear infiltrates (**Figure 5B**).

In addition, the effect of vaccination on the pro-inflammatory cytokine pattern induced in infected mice was evaluated at 4 days postchallenge by measuring by RT-qPCR the mRNA levels of key cytokines on lung homogenates (**Figure 5C**). Compared to control infected mice or mice immunized with one dose of MVA-S, one dose of MVA-S(3P) induced a significant downregulation of IL-12b, CCL11, IP-10, and IFN-γ mRNA levels (**Figure 5C**).

### One Dose of MVA-S(3P) Triggered in K18-hACE2 Transgenic Mice Higher Titers of S- and RBD-Specific IgGs and Neutralizing Antibodies Against Different SARS-CoV-2 Variants of Concern Than MVA-S

Next, we evaluated SARS-CoV-2-specific humoral responses induced in transgenic K18-hACE2 mice vaccinated with one dose of MVA-S(3P) or MVA-S, after vaccine immunization and postchallenge.

**Figure 6:**
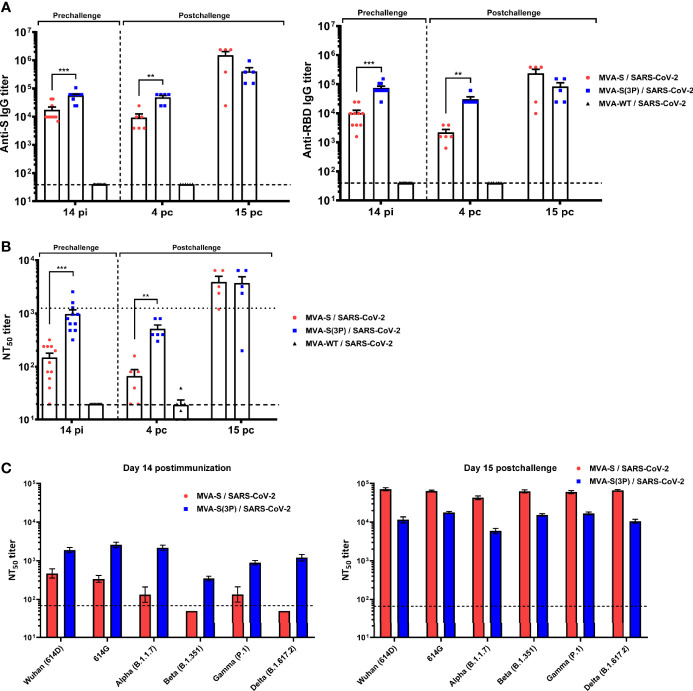
MVA-S(3P) vaccine candidate induced high levels of humoral responses in vaccinated and challenged K18-hACE2 transgenic mice. **(A)** Titers of IgG antibodies specific for the S (left) and RBD (right) proteins (Wuhan strain). Determined by ELISA in individual mouse serum samples collected at day 14 postimmunization (pi) (prechallenge; *n* = 11/group) and at days 4 (*n* = 6/group) and 15 (*n* = 5/group) postchallenge (pc) from K18-hACE2 mice. Mean values and SEM are represented. Dashed line represents the limit of detection. **(B)** SARS-CoV-2 neutralizing antibody titers. NT_50_ titers were evaluated in individual mouse serum samples collected at day 14 pi and at days 4 and 15 pc, using a live virus microneutralization assay (MAD6 strain, having D614G mutation). Mean NT_50_ values and SEM are represented. Upper dotted line represents the levels obtained with the NIBSC 20/136 international standard plasma (containing pooled plasma obtained from 11 individuals recovered from SARS-CoV-2 infection). Bottom dotted line represented the limit of detection. **(C)** SARS-CoV-2 neutralizing antibody titers against SARS-CoV-2 VoC. NT_50_ titers were evaluated in pooled mouse serum samples collected at days 14 pi (left) and 15 pc (right), using VSV-based pseudoparticles expressing the SARS-CoV-2 S protein of different VoC. Mean NT_50_ values and 95% confidence intervals are represented. Dashed line represents the limit of detection. Student’s *t*-test: **p < 0.005; ***p < 0.001.

K18-hACE2 mice immunized with one dose of MVA-S(3P) induced significantly higher titers of S- and RBD-specific IgG antibodies at 14 days post immunization than MVA-S-vaccinated mice (**Figure 6A**). Similar differences were detected 4 days after SARS-CoV-2 infection; nonetheless, the two vaccinated groups showed comparable IgG titers against S and RBD at day 15 postchallenge (**Figure 6A**), reflecting an expansion effect due to the infection, which was more considerable in the MVA-S-vaccinated group.

Analysis of IgG isotypes against S and RBD proteins at the prechallenge and postchallenge time points showed superior titers of IgG2c than IgG1 and lower titers of IgG3 antibodies in both immunization regimens, leading to a IgG2c/IgG1 ratio higher than 1 (**Table 1**), which is indicative of a Th1-type protective humoral responses.

Consistently and similarly to IgG antibody titers, one dose of MVA-S(3P) induced significantly higher titers of neutralizing antibodies against live SARS-CoV-2 than MVA-S at 14 days postimmunization and at 4 days postchallenge, although at day 15 postchallenge, the neutralization titers were enhanced and similar in both groups (**Figure 6B**), reflecting again the existence of a breakthrough infection.

Remarkably, at 14 days postimmunization, similar neutralizing antibody titers against SARS-CoV-2 parental Wuhan strain, D614G mutant, and the VoC alpha (B.1.1.7), gamma (P.1), and delta (B.167.2) were detected in serum from mice immunized with MVA-S(3P), markedly higher than the titers in MVA-S-vaccinated mice (**Figure 6C**, left). Nonetheless, the beta (B.1.351) VoC was neutralized poorly with all the serum samples (**Figure 6C**, left), with 5.44-fold and 9.36-fold reduction of neutralization versus the parental Wuhan strain in serum samples from MVA-S(3P)- and MVA-S-vaccinated mice, respectively (**Supplementary Table 1**). SARS-CoV-2 infection markedly boosted neutralization titers against the Wuhan strain, D614G mutant, and all VoC at day 15 postchallenge in both vaccinated groups, but titers induced by MVA-S vaccination were higher than those elicited by MVA-S(3P), suggesting that MVA-S(3P) vaccination controlled the infection better (**Figure 6C**, right). Moreover, after challenge, neutralization against the parental Wuhan strain and the different VoC was similar in serum samples from MVA-S(3P)- and MVA-S-vaccinated mice (**Figure 6C**, right; **Supplementary Table 1**).

## Discussion

MVA vectors have shown excellent immunogenicity and efficacy in different animal models (33, 34) and parental MVA has been approved by FDA and EMA as a vaccine against smallpox and monkeypox; also, recombinant MVA-BN-FILO has been approved as a combined vaccine against Ebola virus (35). Previous recent studies with MVA vectors expressing S protein have shown good immunogenicity and efficacy results against SARS-CoV-2 in mice, hamsters, and monkey models (10–15). These studies were performed with MVA-based vaccine candidates expressing either a non-stabilized S protein (10, 11, 15) or a prefusion-stabilized S protein (13, 14).

The prefusion state of the SARS-CoV-2 S protein, which maintains the most neutralization-sensitive epitopes, is generally transient and metastable, as success of the infection depends on structural rearrangement in the S that is initiated by virus binding to its entry ACE2 receptor and leads to virus–cell membrane fusion (36). Therefore, to preserve the virus envelope S trimer conformation in vaccine candidates, preventing its transition to fusogenic conformation is likely to facilitate the generation of neutralizing antibodies. For example, in the human immunodeficiency virus (HIV-1) Env protein, the combination of proline insertions in the gp41 subunit and interdomain disulfide bonds successfully enabled trimer stabilization and rational vaccine development (37). Similar results reported with Middle East respiratory syndrome coronavirus (MERS-CoV) (38), SARS-CoV (39), and SARS-CoV-2 (13, 40, 41) demonstrated that mutations to proline of two consecutive residues in the S2 subunit between the central helix and heptad repeat 1 domains stabilized the S protein in a prefusion conformation, conferring resistance to conformational changes induced by receptor recognition or proteolysis and, therefore, increasing S-induced immune responses. In particular, it has been observed in transgenic K18-hACE2 mice full efficacy against SARS-CoV-2 with a single dose of an MVA recombinant expressing an S protein with furin site mutated, two mutations to prolines in the S2 region, and removal of part of C-terminus of the S protein (13). Similarly, substitution of 6 amino acids for prolines in the S protein of SARS-CoV-2 enhanced the immunogenicity of a recombinant Newcastle disease vector vaccine (42).

In the present study, we aimed to enhance the SARS-CoV-2 S-specific immune responses within the poxvirus MVA vaccine platform by generating a novel optimized MVA vector, termed MVA-S(3P), expressing a prefusion-stabilized S(3P) protein, which lacked the furin cleavage site between S1 and S2 subunits and contained three amino acid substitutions to proline (P942, P986, and P987) in the S2 that are known to stabilize prefusion S trimers and enhance S expression (16). We previously confirmed that a purified soluble S form with these 3 prolines was expressed in cultured cells at higher levels than a 2P mutant, and cryo-electron microscopy revealed S(3P) trimers in its prefusion conformation (to be published elsewhere). Here, by Western blot, in non-permissive human HeLa cells infected with MVA-S(3P), we confirmed that expression of S(3P) protein was enhanced by twofold overexpression of S from cells infected with MVA-S. This enhancement was also observed by flow cytometry analysis in infected HeLa cells. By immunofluorescence microscopy, the S(3P) protein was expressed on the surface of cells infected with the recombinant MVA-S(3P) as the native S produced by MVA-S.

It has been reported that enhancing the expression levels of an antigen by recombinant MVA-based vaccine candidates results in a further increase of the antigen-specific immunogenicity (43–45). Thus, the increased levels of S(3P) protein expression are likely responsible for the observed induction of higher titers of IgG antibodies against S and RBD proteins in both wild-type C57BL/6 and transgenic K18-hACE2 mice immunized with one dose of MVA-S(3P), as well as of the higher levels of neutralizing antibodies against live parental SARS-CoV-2 virus that also cross-neutralized different VoC. The recognition of several VoC, including the currently circulating delta variant, highlighted the potent and wide spectrum of humoral responses elicited by MVA-S(3P), a relevant finding as control of COVID-19 progression is mainly mediated by neutralizing antibodies (46–48).

Remarkably, this enhancement in SARS-CoV-2-specific humoral immune responses triggered by MVA-S(3P), in comparison to MVA-S, was translated into higher efficacy against SARS-CoV-2 infection in transgenic K18-hACE2 mice immunized with one dose of MVA-S(3P). This higher efficacy was characterized by the control in body weight and virus replication after SARS-CoV-2 infection, prevention of mortality, less extensive and less severe lung inflammatory lesions, and reduced levels of several pro-inflammatory cytokines that cause lung pathology in severe COVID-19 (49, 50). The induction of all these immune and virological parameters by vaccine candidates have been correlated with SARS-CoV-2 protection and control of COVID-19 progression (14, 30, 46–49, 51–53).

Besides neutralizing antibodies targeting SARS-CoV-2 S protein, other immune responses might also contribute to protection against SARS-CoV-2 infection, as even patients without measurable neutralizing antibodies are able to recover from SARS-CoV-2 infection (54, 55). T-cell responses constitute an additional assistance to control virus spread and restrict virus transmission (56, 57). Here, our results demonstrated that immunization of C57BL/6 mice with two doses of vaccine candidates MVA-S(3P) and MVA-S induced similar strong, polyfunctional, and effector memory adaptive SARS-CoV-2 S-specific CD4^+^ and CD8^+^ T-cell immune responses that may provide additional protection against SARS-CoV-2 infection, beyond other vaccine approaches that mainly induced antibody responses, such as inactivated virus vaccines or protein-based vaccines (58, 59). Moreover, a predominant induction of a Th1-type immune response has been observed in both C57BL/6 and transgenic K18-hACE2 mice vaccinated with either one or two doses of MVA-S and MVA-S(3P), reflecting again the robust immunogenicity elicited by these vaccine candidates; strong Th1 cell response has also been associated with less severe cases of COVID-19, whereas Th2 cell responses correlated with more severe lung disease in humans (60).

Thus, the strength of the SARS-CoV-2-specific immune responses conferred by a single dose of MVA-S(3P), as well as the robust protection induced, provided an important breakthrough in poxvirus vaccine development, based on the preclinical safety, immunogenicity, and efficacy of this vaccine candidate. Overall, the MVA-S(3P) vaccine candidate fulfills the requirements as a vaccine against SARS-CoV-2 infection, with the possibility of usage as a single-dose administration in clinical trials, either alone or in combination with other vaccines.

## Data Availability Statement

The original contributions presented in the study are included in the article/**Supplementary Material**. Further inquiries can be directed to the corresponding authors.

## Ethics Statement

The immunogenicity and efficacy animal studies were approved by the Ethical Committee of Animal Experimentation (CEEA) of the CNB (Madrid, Spain) and by the Division of Animal Protection of the Comunidad de Madrid (PROEX 49/20, 169.4/20, and 161.5/20). Animal procedures conformed with international guidelines and with Spanish law under the Royal Decree (RD 53/2013).

## Author Contributions

Conceptualization: JG-A and ME. Formal analysis: PP, AL-F, and JG-A. Funding acquisition: JG-A, JC, RD, and ME. Investigation: PP, AL-F, CZ, PS-C, DA, JL, RD, and JG-A. Methodology: PP, AL-F, CZ, PS-C, JL, RD, and JG-A. Resources: JC and RD. Supervision: JG-A and ME. Validation: PP, AL-F, and JG-A. Visualization: PP, AL-F, and JG-A. Writing—original draft: JG-A, PP, AL-F, and ME. Writing—review and editing: all authors. All authors contributed to the article and approved the submitted version.

## Funding

This research was supported by Spanish Health Ministry, Instituto de Salud Carlos III (ISCIII), Fondo COVID-19 grant COV20/00151, Fondo Supera COVID-19 (Crue Universidades-Banco Santander) grant and Spanish Research Council (CSIC) grant 202120E079 (to JG-A), CSIC grant 2020E84, La CaixaImpulse grant CF01-00008, Ferrovial and MAPFRE donations (to ME), and Spanish Ministry of Science and Innovation (MICINN), Spanish Research Agency grant PID2020-114481RB-I00 (to JG-A and ME). This research work was also funded by the European Commission-NextGenerationEU, through CSIC’s Global Health Platform (PTI Salud Global) (to JG-A and ME). JC acknowledges MICINN and CSIC support (project number 202020E079). RD received grants from the European Commission Horizon 2020 Framework Programme: Project VIRUSCAN FETPROACT-2016: 731868 and EPIC-CROWN-2-2021:101046084, and Fundación Caixa-Health Research HR18-00469 (Project StopEbola).

## Conflict of Interest

The authors declare that the research was conducted in the absence of any commercial or financial relationships that could be construed as a potential conflict of interest.

## Publisher’s Note

All claims expressed in this article are solely those of the authors and do not necessarily represent those of their affiliated organizations, or those of the publisher, the editors and the reviewers. Any product that may be evaluated in this article, or claim that may be made by its manufacturer, is not guaranteed or endorsed by the publisher.
