# Supplementary material for: A Single Dose of an MVA Vaccine Expressing a Prefusion-Stabilized SARS-CoV-2 Spike Protein Neutralizes Variants of Concern and Protects Mice From a Lethal SARS-CoV-2 Infection
Source: Front Immunol. 2022 Jan 27;12:824728. doi: 10.3389/fimmu.2021.824728 (PMC8829548; doi:10.3389/fimmu.2021.824728)
Supplement: Supplementary file 1 [file Presentation_1.pptx]

## Slide 1
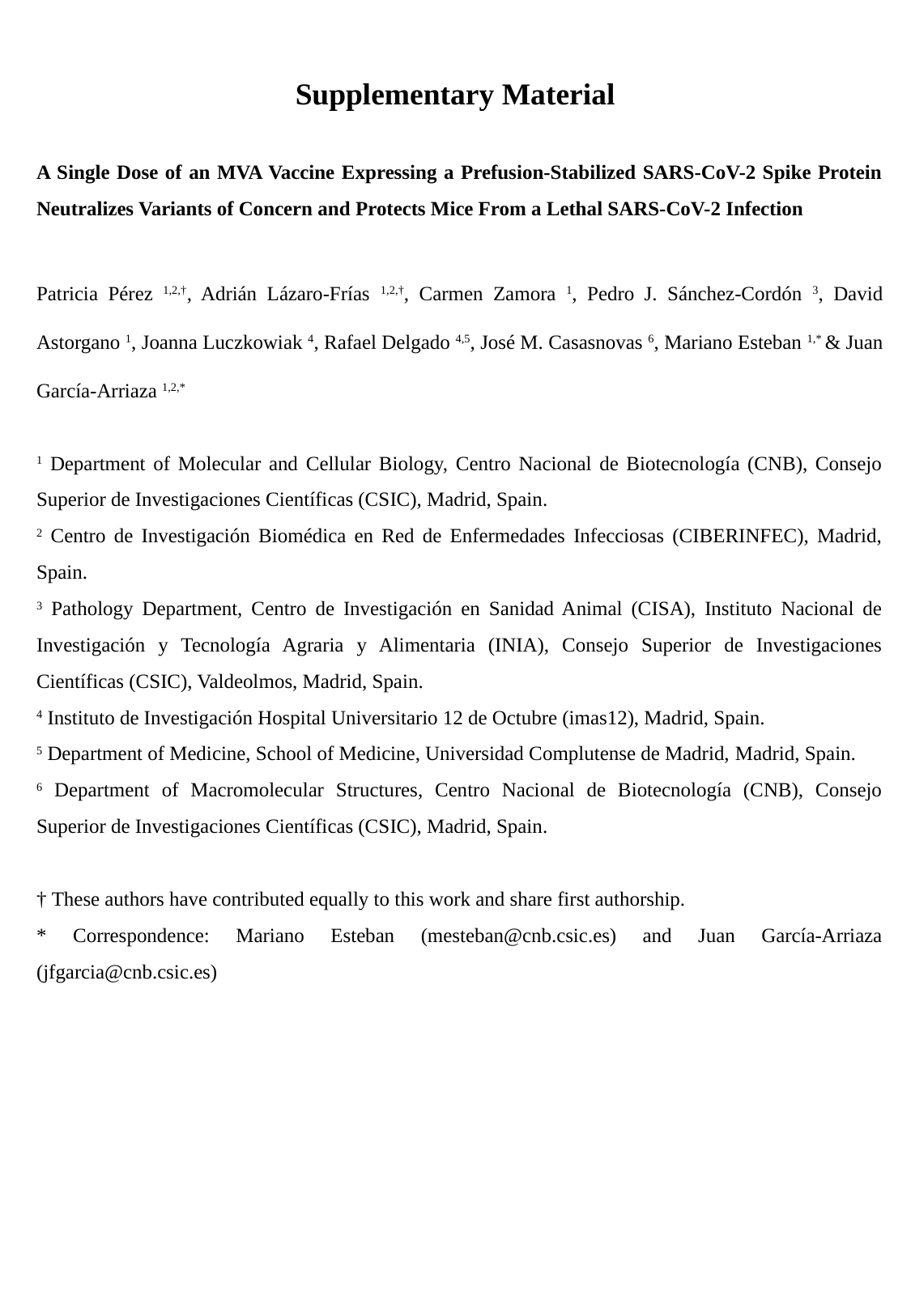

Supplementary Material
A Single Dose of an MVA Vaccine Expressing a Prefusion-Stabilized SARS-CoV-2 Spike Protein Neutralizes Variants of Concern and Protects Mice From a Lethal SARS-CoV-2 Infection
Patricia Pérez 1,2,†, Adrián Lázaro-Frías 1,2,†, Carmen Zamora 1, Pedro J. Sánchez-Cordón 3, David Astorgano 1, Joanna Luczkowiak 4, Rafael Delgado 4,5, José M. Casasnovas 6, Mariano Esteban 1,* & Juan García-Arriaza 1,2,*
1 Department of Molecular and Cellular Biology, Centro Nacional de Biotecnología (CNB), Consejo Superior de Investigaciones Científicas (CSIC), Madrid, Spain.
2 Centro de Investigación Biomédica en Red de Enfermedades Infecciosas (CIBERINFEC), Madrid, Spain.
3 Pathology Department, Centro de Investigación en Sanidad Animal (CISA), Instituto Nacional de Investigación y Tecnología Agraria y Alimentaria (INIA), Consejo Superior de Investigaciones Científicas (CSIC), Valdeolmos, Madrid, Spain.
4 Instituto de Investigación Hospital Universitario 12 de Octubre (imas12), Madrid, Spain.
5 Department of Medicine, School of Medicine, Universidad Complutense de Madrid, Madrid, Spain.
6 Department of Macromolecular Structures, Centro Nacional de Biotecnología (CNB), Consejo Superior de Investigaciones Científicas (CSIC), Madrid, Spain.
† These authors have contributed equally to this work and share first authorship.
* Correspondence: Mariano Esteban (mesteban@cnb.csic.es) and Juan García-Arriaza (jfgarcia@cnb.csic.es)

## Slide 2
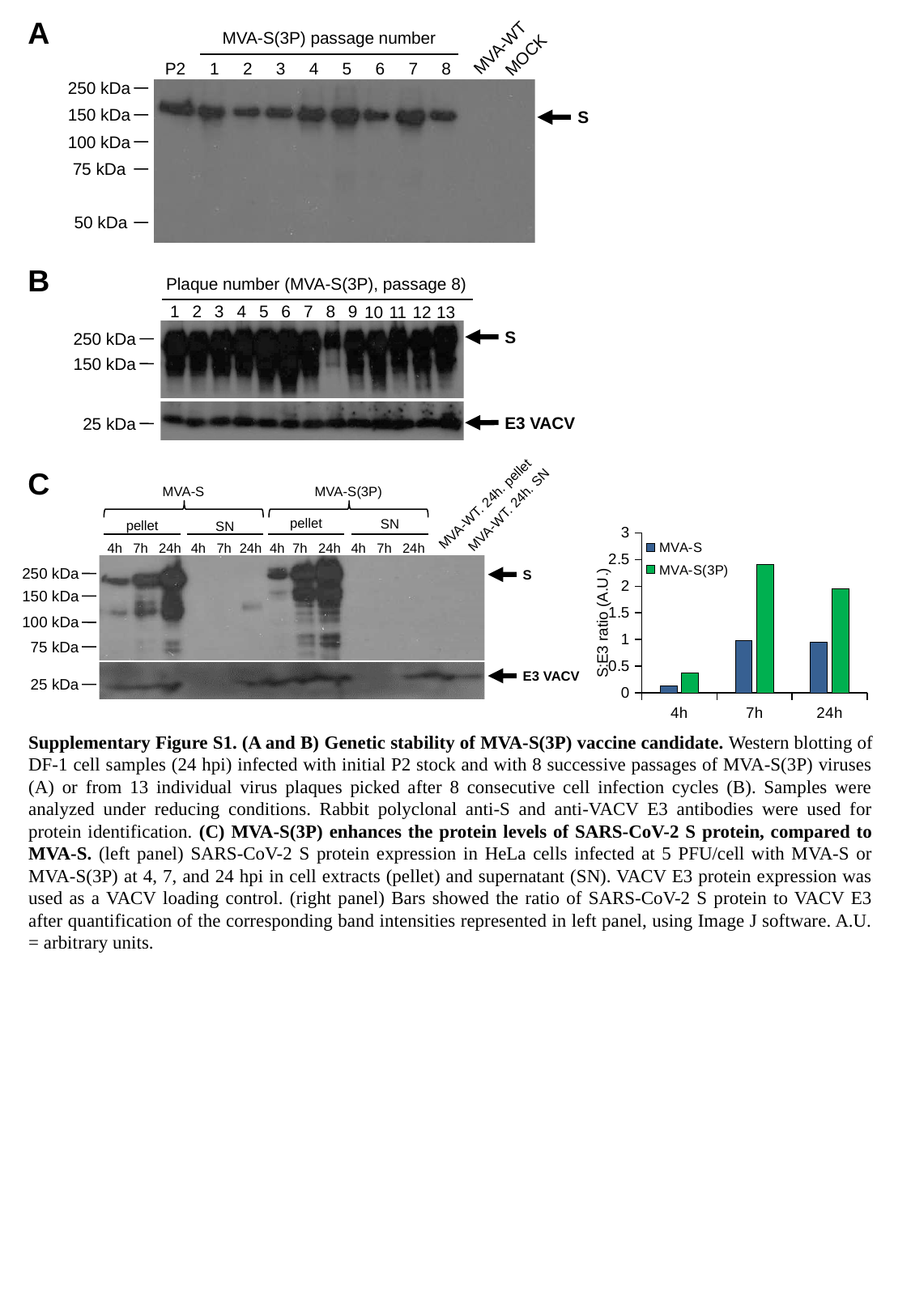

A
MVA-S(3P) passage number
MVA-WT
MOCK
P2
1
2
3
4
5
6
7
8
250 kDa
150 kDa
S
100 kDa
75 kDa
50 kDa
B
Plaque number (MVA-S(3P), passage 8)
1
2
3
4
5
6
7
8
9
10
11
12
13
S
250 kDa
150 kDa
E3 VACV
25 kDa
C
MVA-S
MVA-S(3P)
MVA-WT. 24h. pellet
MVA-WT. 24h. SN
pellet
SN
pellet
SN
### Chart
| Category | | |
|---|---|---|
| 4h | 0.12771183308005093 | 0.37498859195712236 |
| 7h | 0.9792057166923529 | 2.400201720668002 |
| 24h | 0.9544711852964809 | 1.9489523128188715 |4h
7h
24h
4h
7h
24h
4h
7h
24h
4h
7h
24h
250 kDa
S
150 kDa
100 kDa
75 kDa
E3 VACV
25 kDa
Supplementary Figure S1. (A and B) Genetic stability of MVA-S(3P) vaccine candidate. Western blotting of DF-1 cell samples (24 hpi) infected with initial P2 stock and with 8 successive passages of MVA-S(3P) viruses (A) or from 13 individual virus plaques picked after 8 consecutive cell infection cycles (B). Samples were analyzed under reducing conditions. Rabbit polyclonal anti-S and anti-VACV E3 antibodies were used for protein identification. (C) MVA-S(3P) enhances the protein levels of SARS-CoV-2 S protein, compared to MVA-S. (left panel) SARS-CoV-2 S protein expression in HeLa cells infected at 5 PFU/cell with MVA-S or MVA-S(3P) at 4, 7, and 24 hpi in cell extracts (pellet) and supernatant (SN). VACV E3 protein expression was used as a VACV loading control. (right panel) Bars showed the ratio of SARS-CoV-2 S protein to VACV E3 after quantification of the corresponding band intensities represented in left panel, using Image J software. A.U. = arbitrary units.

## Slide 3
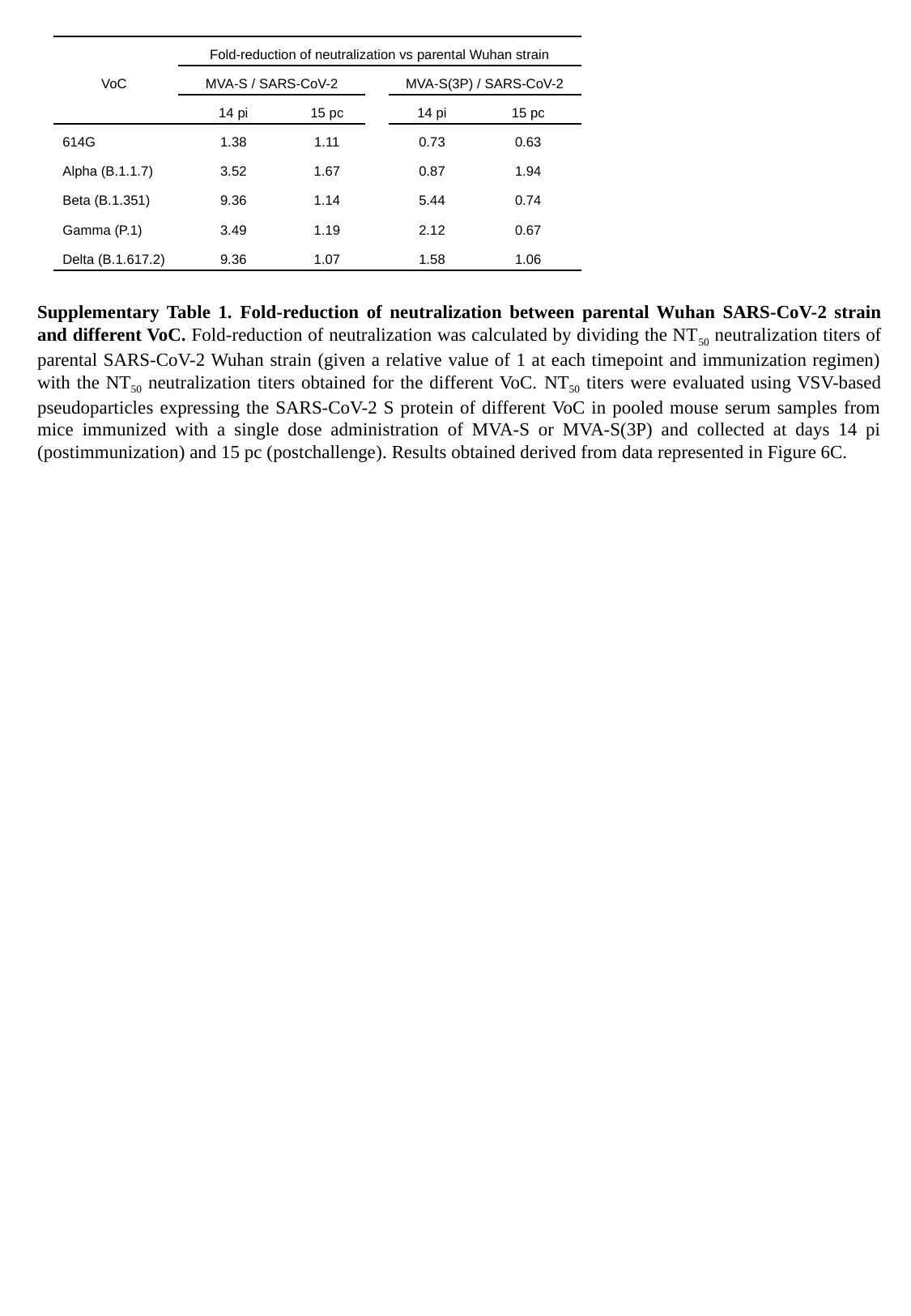

| VoC | Fold-reduction of neutralization vs parental Wuhan strain | | | | |
| --- | --- | --- | --- | --- | --- |
| | MVA-S / SARS-CoV-2 | | | MVA-S(3P) / SARS-CoV-2 | |
| | 14 pi | 15 pc | | 14 pi | 15 pc |
| 614G | 1.38 | 1.11 | | 0.73 | 0.63 |
| Alpha (B.1.1.7) | 3.52 | 1.67 | | 0.87 | 1.94 |
| Beta (B.1.351) | 9.36 | 1.14 | | 5.44 | 0.74 |
| Gamma (P.1) | 3.49 | 1.19 | | 2.12 | 0.67 |
| Delta (B.1.617.2) | 9.36 | 1.07 | | 1.58 | 1.06 |
Supplementary Table 1. Fold-reduction of neutralization between parental Wuhan SARS-CoV-2 strain and different VoC. Fold-reduction of neutralization was calculated by dividing the NT50 neutralization titers of parental SARS-CoV-2 Wuhan strain (given a relative value of 1 at each timepoint and immunization regimen) with the NT50 neutralization titers obtained for the different VoC. NT50 titers were evaluated using VSV-based pseudoparticles expressing the SARS-CoV-2 S protein of different VoC in pooled mouse serum samples from mice immunized with a single dose administration of MVA-S or MVA-S(3P) and collected at days 14 pi (postimmunization) and 15 pc (postchallenge). Results obtained derived from data represented in Figure 6C.
